# Review on the Impact of Milk Oligosaccharides on the Brain and Neurocognitive Development in Early Life

**DOI:** 10.3390/nu15173743

**Published:** 2023-08-26

**Authors:** Yuting Fan, Arden L. McMath, Sharon M. Donovan

**Affiliations:** 1 Department of Food Science and Human Nutrition, University of Illinois Urbana-Champaign, Urbana, IL 61801, USA; yutingf2@illinois.edu; 2 Division of Nutritional Sciences, University of Illinois Urbana-Champaign, Urbana, IL 61801, USA; amcmath2@illinois.edu

**Keywords:** narrative review, milk oligosaccharides, HMO, cognition, neurodevelopment, brain development, early life

## Abstract

Milk Oligosaccharides (MOS), a group of complex carbohydrates found in human and bovine milk, have emerged as potential modulators of optimal brain development for early life. This review provides a comprehensive investigation of the impact of milk oligosaccharides on brain and neurocognitive development of early life by synthesizing current literature from preclinical models and human observational studies. The literature search was conducted in the PubMed search engine, and the inclusion eligibility was evaluated by three reviewers. Overall, we identified 26 articles for analysis. While the literature supports the crucial roles of fucosylated and sialylated milk oligosaccharides in learning, memory, executive functioning, and brain structural development, limitations were identified. In preclinical models, the supplementation of only the most abundant MOS might overlook the complexity of naturally occurring MOS compositions. Similarly, accurately quantifying MOS intake in human studies is challenging due to potential confounding effects such as formula feeding. Mechanistically, MOS is thought to impact neurodevelopment through modulation of the microbiota and enhancement of neuronal signaling. However, further advancement in our understanding necessitates clinical randomized-controlled trials to elucidate the specific mechanisms and long-term implications of milk oligosaccharides exposure. Understanding the interplay between milk oligosaccharides and cognition may contribute to early nutrition strategies for optimal cognitive outcomes in children.

## 1. Introduction

Cognition refers to complex mental functions, including memory, learning, thinking, and perception [1]. During infancy and early childhood, brain growth and the development of cognitive, behavioral, and social functions occurs rapidly [2] and influence later academic achievement [3]. Although cognitive development in this critical early life period is impacted by numerous factors [4], nutrition is at the forefront [4]. Previous research suggests that suboptimal nutrition early in life is associated with poorer cognitive development and functioning [5]. For example, nutritional supplementations for infants and young children led to a significant increase in cognition and school performance later in life [6]. 

Human milk is the optimal nutrition source for infants [7]. The American Academy of Pediatrics recommends exclusive breastfeeding for the first six months of life and 12 months or longer in combination with complementary foods [8]. Breastfeeding is associated with various benefits, such as preventing infectious disease, reducing all-cause mortality, and influencing both short-term and long-term infant development [9]. Specifically, feeding preterm and term infants human milk rather than formula has demonstrated benefits for neurodevelopment [10], an effect that may persist through adolescence [11,12,13,14]. The benefits of human milk for neurodevelopment have been attributed to components of its complex composition [15], including a group of complex carbohydrates known as human milk oligosaccharides (HMOs) [16]. While the milk of all mammals contains milk oligosaccharides (MOS), human milk is unique in its high concentration and structural complexity of oligosaccharides [16]. Infant formulas made from bovine milk have historically contained low concentrations of predominantly sialylated oligosaccharides compared to human milk [17,18,19], although one or two to up to five or six different synthetic MOS have been added to some infant formulas [20,21,22].

Numerous associations between HMOs and neurodevelopment and cognitive outcomes have been reported [23]. The gut microbiota has been shown to play an essential role in this interaction [24,25] through the gut-brain axis, defined as bidirectional communication between the enteric and central nervous systems [24]. HMOS exhibit physiological functions related to establishing healthy microbiota during early life [26] and may promote brain and cognitive development through the gut-brain axis. However, more than 200 unique HMOs structures have been identified [16], making it difficult to understand the unique function of individual HMOs with different structures on the neurocognitive development of infants from observational studies. 

The benefits of HMOs for cognitive development may be due to their components, sialic acid and fucose, which have been implicated in brain development. Sialic acid (SA) is an essential nutrient for brain development as it is a component of gangliosides in the brain [27]. Also, SA has been reported to be an essential compound during neurodevelopment as it supports brain development, neuro transmission, and synapse formation [28]. 

SA rarely exist in free form in nature [29]; in milk, it is bound to lactose to form sialyllactose (SL) to more complex oligosaccharides or glycoproteins [30]. SL is the most abundant MOS in bovine milk, although its concentration is still lower than in human milk [31]. Accordingly, the SL concentration in bovine-milk-based infant formula is lower than that of human milk [32]. Fucosylated oligosaccharides predominate in human milk, such as fucosyllactose (FL) and HMOs, with more complex structures, whereas bovine milk contains very low fucosylated oligosaccharides [33]. Fucosylated glycans are implicated in neuronal processes that underpin neuronal development, learning and memory [34,35,36,37]. The composition of human milk differs by secretor genotypes, where secretor mothers have a functional *FUT2* gene that produces α1,2-fucosylated compounds, with 2′-FL being the most abundant [38]. However, preclinical studies suggested that intact 2′-FL does not cross the blood-brain barrier, although cleaved fucose or other gut microbial 2′-FL metabolites may be incorporated into the brain [39,40]. Thus, this raises the question of whether the effects of MOS were mediated directly via the incorporations of MOS components in the brain or indirectly via the mediation of microbiota and gut-brain axis.

The goals of this review are to critically appraise the current evidence from preclinical and human studies relating to MOS and neurocognitive outcomes, discuss potential mechanisms of action, identify key knowledge gaps, and highlight areas for future research. Although others have reviewed HMOS’s role in infant cognitive development, they focused on interventions in animal models [41] or observational studies in human infants alone [42]. Since this field is rapidly evolving, we aim to synthesize and critically evaluate the current evidence on the role of MOS, both HMOs and bovine milk oligosaccharides (BMOS), in brain or cognitive development in early life. 

## 2. Materials and Methods

### 2.1. Search Strategy

The literature search was performed in the PubMed database until April 2023. The search terms included * Infant(s), child, children, early childhood, toddler(s), early life, mice, mouse, murine, rat, rodent, piglet, monkey, animal, human milk oligosaccharide, HMO(s), bovine milk oligosaccharide, BMO(s), 2′-fucosyllactose, 3′-sialyllactose, 6′-sialyllactose, sialyllactose, sialylation, fucosylation, sialic acid, cognitive function, cognitive control, cognition, self-regulation, self-control, executive function, inhibition, inhibitory control, attention, fMRI, interference control, working memory, short-term memory, long-term memory, episodic memory, spatial memory, cognitive flexibility, task switching, social, emotion, temperament, negative affect, positive affect, mood, neural development, neural growth, neurogenesis, prefrontal cortex, dorsolateral prefrontal cortex, anterior cingulate cortex, cerebral cortex, hippocampus, amygdala, basal ganglia, striatum, brain, (gastro)intestinal microbiome, (gastro)intestinal microbiota, (gastro)intestinal microbes, gut microbiome, gut microbiota, gut microbes, fecal microbiome, fecal microbiota, fecal microbes, metagenome(s), metabolome(s), metabolite(s), short-chain-fatty acid(s), and volatile fatty acid(s).

### 2.2. Selection Criteria

To be included in the review, studies need to be related to MOS exposure in early life, any aspects of cognitive development, and were either interventions were conducted in animal models or observational studies in human subjects. Search terms related to gut microbiome were also included in the search strategy to identify potential studies about the gut-brain axis. Articles were excluded if there was no full version, no English version, or if it was conducted in adults. All articles were initially reviewed by two authors (YF and ALM) to determine inclusion in the review. In the case of disagreements, the third author (SMD) resolved the discrepancies.

### 2.3. Data Extraction

The data extracted included the authors, year of the publication, study location, experimental design, sample size, intervention, study duration, and relevance to brain and cognitive development, or the gut-brain axis. 

## 3. Results

### 3.1. Study Selection

**Figure 1:**
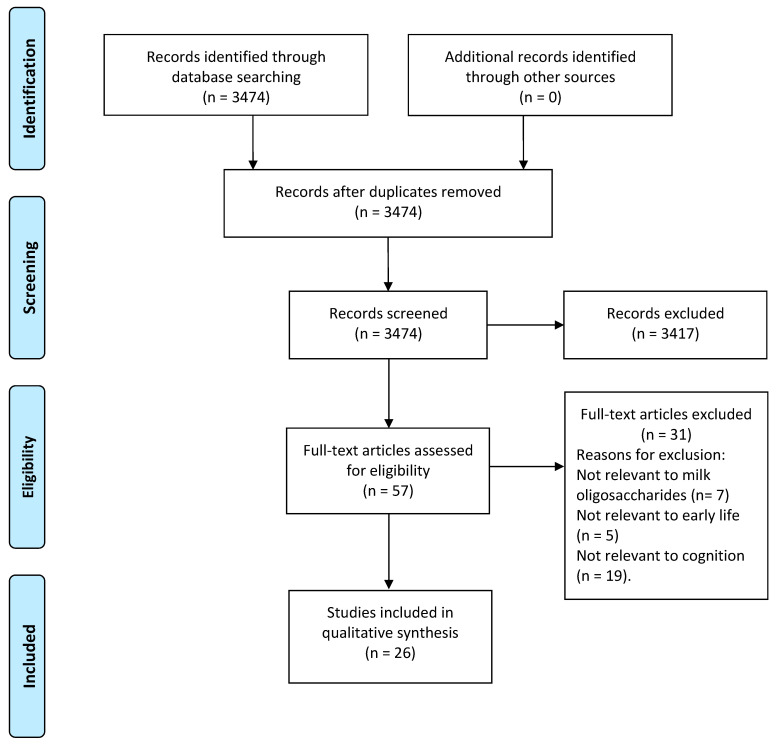
PRISMA flow diagram for the study selection process.

The PRISMA flow diagram [43] is shown in Figure 1. A total of 3474 articles were identified through the PubMed database. During screening by title and abstract, 3417 studies were outside the scope of our review and therefore excluded. In total, 57 studies underwent full-text review. An additional 31 articles were removed for not being relevant to MOS (*n =* 7), not relevant to early life (*n =* 5), and not related to cognition (*n =* 19). As a result, 26 publications met the inclusion criteria, reporting the associations between milk oligosaccharide exposure during the early developmental period and the brain or cognitive development of the offspring.

### 3.2. Study Characteristics

The characteristics of the 26 selected studies are presented in Table 1 and Table 2. Most (69%) studies were intervention studies in animal models (*n =* 18) (Table 1), and the rest were observational studies involving human subjects (*n =* 8) (Table 2). The most common study interventions were milk oligosaccharide supplementation (*n =* 15) to either pig (*n =* 9) or rodent models (*n =* 6). There was a wide variety of methods used for the assessment of cognition and brain development. Magnetic resonance imaging (MRI), magnetic resonance spectroscopy (MRS), and Hippocampal gene expressions were the most common methods for investigating differences in brain structures between control and treatment groups.

Memory and learning in animal models are often accessed through observation of behaviors following a stimulus. The most common behavioral tests for cognitive function in the present review were novel object recognition (NOR), T-maze, and Y-maze. NOR refers to the evaluation of differences in the exploration time of the animal for novel and familiar objects [44,45]. The NOR task contains three phases: habituation, familiarization, and test phase. In habituation, the animals can freely explore an open-field arena without objects. For familiarization, a single animal will be introduced to two identical sample objects in the open field. Lastly, during the test phase, the animals will be returned to the open-field arena, where they encounter a sample project identical to the previous one and a novel one [44]. 

Because there is no reward involved in NOR, animals explore novel objects based on their natural preferences [44]. The increased time spent exploring novel objects reflects greater cognitive skills from the animal subjects [46], and it has been suggested that the dorsal hippocampus plays a vital role in NOR memory formation [47].

T-maze and Y-maze Spontaneous Alternative tests are two similar assessment tools for spatial working and reference memory, the only difference being the shape of the apparatus (Y versus T-shaped apparatus) [48]. Both mazes are based on the natural tendency of animals for preference to explore a novel arm compared to a familiar arm [49]. T-maze and Y-maze assess habit learning and short-term habituation in a novel environment [49,50].

**Table 1:** Characteristics of intervention studies in preclinical animal models.

Ref.	First Author and Year	Country	Subjects	Sample Size (*n*)	Study Duration	Intervention or Exposure
[51]	Tarr, 2015	US	Mice; male C57/BL6	54	Upon arrival for 20 days	Diets: CON: AIN-93G semi-purified laboratory mouse diet 6′-SL: CON + 5% 6′-SL 3′-SL: CON + 5% 3′-SL Groups: Stress (with social disruption stressor) Non-stress
[52]	Jacobi, 2016	US	Pigs	54	21 days	Diets: CON: formula adjusted for nutrient requirements of neonatal pigs CON +2 g 3′-SL/L CON +4 g 3′-SL/L CON +2 g 6′-SL/L CON +4 g 6′-SL/L CON +2 g PDX/L + 2 g GOS/L
[53]	Oliveros, 2016	Spain	Rat pups; Lister Hooded & Sprague-Dawley	60 (Lister Hooded) & 60 (Sprague-Dawley)	PND 3 until weaning	Diets: Control: BF + 1 g/kg body weight of water 2′-FL: BF + 1 g/kg body weight of 2′-FL per day
[54]	Mudd, 2017	US	Pigs; vaginally delivered male	38	PND 2 until PND 32 or 33	Diets: Control (CON): formula for nutritional needs of growing pigs LOW: CON + bovine-derived modified why enriched with SL(SAL-10) (130 mg SL/L) MOD: CON + SAL-10 (380 mg SL/L) HIGH: CON + SAL-10 (760 mg SL/L)
[55]	Oliveros, 2018	Spain	Rats; Sprague-Dawley	30	PND 3 until weaning (PND 22)	Diets: CON: BF + water Neu5AC: BF + free Neu5AC to SA level in rat milk 6′-SL: BF + free 6′-SL to mimic natural SA in rat milk
[56]	Fleming, 2018	US	Pigs; naturally farrowed male	36	PND 2 until PND 22	Diets: CON: formula for nutritional needs of growing pigs Sialyllactose: CON + SAL-10 (380 mg SL/L)
[57]	Obelitz-Ryom, 2019	Denmark	Piglets; male & female	40 (preterm), 14 (term, C-section), 12 (term, vaginal)	PND 0 until PND19	Diets: PRE-CON: Raw bovine milk + 6 g/L lactose PRE-SAL: Raw bovine milk + 8.5 g/L SAL-10 (380 mg SL/L) TERM-CON: Raw bovine milk + 6 g/L lactose TERM-NAT: Naturally BF
[58]	Wang, 2019	Australia	Piglets; domestic *Sus scrofa*	46	PND 3 until PND 38	Diets: CON: sow milk replacer SL: Control + 3′-SL (7.6 g/kg) and 6′-SL (1.9 g/kg) SL/SLN: Control + 3′-SL (7.04 g/kg), 6′-SL (1.74 g/kg), and 6′-sialyllactosamine (0.72 g/kg)
[59]	Lee, 2020	US	Mice; C57/BL6 male	36	Six weeks of age until 12 weeks	Diets: LF: 10% kcal as fat diet HF: 45% kcal as fat diet HF 1% 2′-FL: HF + 98.4% purity 2′-FL 1% (*w/v*) HF 2% 2′-FL: HF + 98.4% purity 2′-FL 2% (*w/v*) HF 5% 2′-FL: HF + 98.4% purity 2′-FL 5% (*w/v*) HF 10% 2′-FL: HF + 98.4% purity 2′-FL 10% (*w/v*)
[60]	Fleming, 2020	US	Pigs; male	36	PND 2 until PND 33	Diets: CON: milk replacer OF: CON + 5 g/L Oligofructose (OF) + 0 g/L 2′-FL OF+2′-FL: CON + 5 g/L OF + 1 g/L 2′-FL
[61]	Fleming, 2020	US	Pigs; male	48	PND 2 until PND 33	Diets: CON: sow milk replacer BMOS ^1^: CON + 12.4 g/L BMOS HMO: CON + 1.0 g/L of 2′-FL + 0.5 g/L of LNnT BMOS + HMO: CON + 12.4 g/L of BMOS + 1.0 g/L of 2′-FL + 0.5 g/L of LNnT
[62]	Tuplin, 2021	Canada	Rats; Sprague-Dawley both sex	40	PND 1 for Eight weeks	Diets: CON: AIN-93G nutritionally complete diet 3′-SL: CON + 0.625% wt/wt 3′-SL 2′-FL: CON + 0.625% wt/wt 2′-FL 3′-SL+2′-FL: CON + 0.625% wt/wt 3′-SL + 0.625% wt/wt 2′-FL
[63]	Pisa, 2021	Italy	Mice; male	28	PND 0 until 23 weeks	Genotypes: WT: wild type KO: knock-out for the gene synthesizing 3′-SL Diets: CTRL: WT offspring with 3′-SL in milk MILK: WT offspring with reduced 3′-SL in milk GENE: KO offspring with 3′-SL in milk GENE + MILK: KO offspring with reduced 3′-SL in milk
[64]	Hauser, 2021	Italy	Mice; male	146	PND 0 until 25 weeks	Genotypes: WT: wild type KO: knock-out for the gene synthesizing 6′-SL Diets: CTRL: WT offspring with 6′-SL in milk MILK: WT offspring without 6′-SL in milk GENE: KO offspring with 6′-SL in milk GENE + MILK: KO offspring without 6′-SL in milk
[65]	Clouard, 2021	Denmark	Göttingen minipigs; female	64	Two weeks until 45 weeks	Diets: Sow-reared: fed porcine milk until weaning. Formula-fed: formula diets until weaning: CON: Milk replacer with no additional oligosaccharides FN: CON + 4 g/L mixture of fucosylated (2′-FL + di-FL) and neutral (LNT + LNnT) oligosaccharides SL: CON + 0.68 g/L sialylated oligosaccharides (3′-SL + 6′-SL) FN + SL: CON + 4 g/L FN + SL After weaning to a high-energy, pelleted, obesogenic diet.
[66]	Lee, 2021	US	Mice; C57BL/6J male	32	Six weeks until 14 weeks	Diets: LF/CON: 10% kcal as fat diet HF/CON: 45% kcal as fat diet LF/2′-FL: LF/CON + 10% 2′-FL (*w/w*) HF/2′-FL: HF/CON + 10% 2′-FL (*w/w*)
[67]	Sutkus, 2022	US	Pigs; male	52	PND 2 until PND 34 or 35	Diets: CON: Milk replacer supplemented with 0.532% lactose FL: Con + 0.532% 2′-FL BI: Con + 109 CFU/pig/d Bi-26 FLBI: FL + BI
[68]	Pisa, 2023	Italy	Mice; both sex	46 (Expt 1) 48 (Expt 2)	PND 0 until 25 weeks	Genotype: WT: wild type dKO: double-knock-out for the genes synthesizing 3′-SL and 6′-SL Diets—Experiment 1: CTRL: WT offspring with WT BF MILK: WT offspring with dKO BF GENE: dKO offspring with WT BF GENE + MILK: KO offspring with dKO BF Diets—Experiment 2: CTRL-H_2_O: WT offspring with WT BF + water MILK-H_2_O: WT offspring with dKO BF + water CTRL-SL: WT offspring with WT BF + 3′SL and 6′SL MILK-SL: WT offspring with dKO BF + 3′SL and 6′SL

^1^ BMOS: Milk oligosaccharides derived from bovine whey, composed of primarily GOS + trace amount of 3′-SL and 6′-SL. Abbreviations: CON, control; PND, postnatal day; BF, breastfeeding; SA, sialic acid; LNT, Lacto-N-tetraose; LNnT, Lacto-N-neotetraose.

**Table 2:** Characteristics of human infant observational studies.

Ref.	First Author and Year	Country	Subjects	Sample Size	Study Duration	Exposure
[69]	Berger, 2020	US	Hispanic mother-term infant dyads (males and females)	50	Milk collection at 1- and 6-months postpartum Cognition assessment at 24 months of age	19 HMO concentrations
[70]	Cho, 2021	US	Mother-term infant dyads (males and females)	99	Milk collection at study visit (infant at 2–25 months-old) Cognition assessment at study visit	Eight HMO concentrations
[71]	Oliveros, 2021	Spain	Normal weight, overweight, obese, and GDM mother-term infant dyads (males and females)	82	Milk collection at 1-month postpartum Cognition assessment at 6 and 18 months-of-age	Two HMO concentrations
[72]	Jorgensen, 2021	Malawi	Mother-term infant dyads (males and females)	659	Milk collection at 6-months postpartum Cognition assessment at 12 and 18 months-of-age	51 HMO relative abundances
[73]	Ferreira, 2021	Brazil	Mother-term infant dyads (males and females)	73	Milk collection at 1-month postpartum Cognition assessment at 1, 6, and 12 months-of-age	19 HMO concentrations
[74]	Rozé, 2022	France	Mother-preterm infants dyads (males and females)	137	Milk collection for seven weeks from birth Cognition assessment at two years of age	24 HMO and total sialic acid concentrations for mean impute values over samples from 7 weeks
[75]	Berger, 2022	US	Mother-term infant dyads (males and females)	20	Milk collection at one month postpartum MRI scanning at one month postpartum for infants	19 HMO concentrations
[76]	Willemsen, 2023	The Netherlands	Mother-term infant dyads (males and females)	63	Milk collection at 2, 6 and 12 weeks postpartum Cognition assessment at three years of age	24 HMO concentrations

Abbreviations: HMO, human milk oligosaccharides; GDM, gestational diabetes mellitus; MRI: magnetic resonance imaging.

### 3.3. Sialyllactose and Cognition

#### 3.3.1. Term and Preterm Piglet Models

Six studies conducted intervention trials in piglets, analyzing the relationships between the milk oligosaccharide or SL intake on brain and/or cognitive developmental outcomes (Table 3). HMOS have been associated with increased SA delivery to the brain during development [27,77]. Among the sialylated HMOS, SL supplementation was most commonly investigated [31]. The most abundant SL are 6′-SL and 3′-SL [32], which are constitutional isomers sialylated by either α-(2,3) linkage(3′-SL) or α-(2,6) linkage(6′-SL); these two structures were reported to have similar biological functions [78].

Piglets are widely used as a preclinical model for human gut-brain-axis studies [79] due to their similarities with human gastrointestinal physiology [80] and brain development [81]. Only one study utilized preterm piglets to study the influence of dietary SL on cognition, finding that preterm piglets supplemented with SL were more likely to succeed in the learning criteria than the control group [57]. Although supplementation with SL did not directly affect the SA levels in the hippocampus of the preterm piglets, SL supplementation resulted in the up-regulation of the myelination-responsible genes, myelin-associated glycoprotein, myelin basic protein, and genes related to SA metabolism. However, the selected genes involved in memory formation and learning processes were not modified by SL enrichment [57].

Due to the lack of pure forms of synthesized SL, some previous studies have administered a bovine milk extract enriched in 3′-SL and 6′-SL (SAL) (43,45). For instance, in male term piglets, SAL was supplemented in varying amounts: control (55 mg SL/L), low (159 mg SL/L), moderate (429 mg SL/L), or high (779 mg SL/L) from postnatal day 2 through 32 or 33 [54]. The moderate SAL concentration group was selected to represent a concentration similar to human milk [54]. Higher levels of bound SA were found in the prefrontal cortex of the control and high groups compared to the low and moderate groups. Additionally, the moderate group showed a higher ratio of free-to-bound SA in the hippocampus than the control and high-concentration groups. As for brain structures, white matter maturation and the level of axonal tract integrity were measured with diffusion tensor imaging. Increased measures of corpus callosum mean diffusivity (MD), axial diffusivity (AD), and radial diffusivity (RD) were observed in the group with moderate SL supplementation compared to other levels of SAL supplementations and control [54]. Tract-based spatial statistics (TBSS) analysis revealed that the moderate SL group showed higher RD measures in the white matter of the left corpus callosum compared to the low SL group. This study suggests that the effects of SL supplementation are dose-dependent and specific to brain regions, including the corpus callosum, prefrontal cortex, and hippocampus [54].

Piglets receiving the SAL concentration comparable to human milk concentrations exhibited better effects on brain structural development. Similarly, Jacobi et al. compared the different SL isomer supplementation on full-term pigs from day 1 to 21 days. The left hemispheres of the pigs were divided into four regions, and their sialic acid concentrations were measured. The total incorporation of ganglioside-bound SA in the corpus callosum was higher for pigs supplemented with 3′-SL and 6′-SL than the control diet group. Ganglioside-bound SA was also increased in the cerebellum for supplementation with 3′-SL [52]. The corpus callosum is the largest white matter structure in the brain, containing diverse intra- and interhemispheric myelinated axonal projections [52,82]. The impact of dietary SL on the amount of ganglioside-bound SA in the corpus callosum suggests SL may play a role in supporting axonal myelination.

To investigate the effects of sialylated HMO on brain development, another piglet study explored the effects of supplementation of pure 3′-SL and 6′-SL or combined supplementation of pure forms of 3′-SL, 6′-SL, and 6′-sialyl-lactosamine (SLN) compared to a control group. At postnatal day 38, absolute concentrations of 33 brain metabolites were measured, and results showed that sialylated HMO supplementation significantly increased several important brain metabolites and neurotransmitters compared to the control group [58]. For example, with the combination of SL and SLN, the absolute and relative levels of glutamate were significantly increased (*p <* 0.05) compared to control. Thus, this study provides evidence that orally administered sialylated HMOS up-regulated brain levels of glutamate (Glu), which is one of the major excitatory neurotransmitters in the brain that has been suggested to support brain development such as influencing neurite sprouting, synaptogenesis and dendrite pruning [58]. 

Several studies investigated behavioral outcomes in piglets administered HMOS as measures of cognitive development. Fleming et al. reported that supplementation of SL to term male pigs (*n =* 38) did not affect exploratory behaviors, including time spent investigating objects, the number of object visits, or the mean time spent per object visit [56]. Clouard et al. [65] was the only study to compare the effects of sialylated, neutral, and fucosylated HMOS on cognitive outcomes. Dietary treatment of female Göttingen minipigs with HMOS between 2 and 11 weeks of age resulted in significant improvement in behavioral tasks as indicated by spending longer time in the trials compared to the control group. Also, reference memory was increased with SL supplementation compared to control [65]. However, the significant beneficial effects of dietary HMOS were time-dependent, where working and reference memory scores were found to be greater with SL treatment between 16–29 weeks of age (after HMOS treatment had ended) but not at 39–45 weeks. This period (between 16–29 weeks) is the time between weaning and sexual maturity, sometimes referred to as the adolescence age. The time-dependent effects observed in the study raise intriguing questions about the underlying mechanisms of HMOS and their interaction with neural circuits during critical periods of development. The findings also imply that the benefits of HMOS supplementation might have the most pronounced effects during the adolescent period.

#### 3.3.2. Rodent Models

Four studies used young mice to investigate the effects of sialylated milk oligosaccharide exposure in early life on cognitive outcomes (Table 4). Oliveros et al. used Sprague-Dawley rats (*n =* 47) to assess the impact of sialic acid and sialylated oligosaccharides supplementation from birth to postnatal day three on learning and memory outcomes. In the NOR test that evaluates the time rats explored a novel object compared to a familiar object, rats that received sialic acid supplementation in the form of both SA and 6′-SL spent more time exploring the novel object, demonstrating better cognitive abilities [55]. Long-term potentiation (LTP) for signal transmission between neurons, Y maze test and IntelliCage^®^ Protocol test (an automated testing system for spontaneous and learning behavior of rodents) performance at one year were significantly improved with both Neu5Ac and 6′-SL supplementations during lactation [55]. 

Tarr et al. [51] explored the effects of HMO supplementation on the gut microbiota community to investigate the impact of HMO on the gut-brain axis. To assess the potential role of HMOS supplementation in reducing anxiety behaviors, a social disruption stressor was given to the experimental animals for all groups. Supplementations of 5% 3′-SL and 6′-SL supported the maintenance of both normal behaviors and the number of immature neurons in the dentate gyrus under stress compared to the control group. Since it has been proven that immature neurons are important in anxiety-like behaviors and learning, the maintenance of the number of immature neurons by SL may explain the maintenance of normal behavior under stress. Additionally, the microbial community was not significantly affected by stressor exposure when 3′-SL and 6′-SL were supplied in the diet compared to the control group. These results suggest that MOS may support normal behavior under stress conditions by modulating gut microbiota and gut-brain signaling [51]. These findings contribute to the growing understanding of the complex interactions between the gut and the brain and highlight the potential role of HMOS in promoting mental well-being.

Instead of supplementing SL, three studies from the same laboratory used knock-out preclinical mice models to test the effects of sialylated MOS deficiencies [63,64,68]. The first study, Pisa et al. produced knockout (KO) dams that lack the gene that encodes α2,3-sialyltransferase for synthesizing 3′-SL in the mouse mammary gland, achieving around an 80% reduction in 3′-SL content in the milk provided to the pups [63]. Hauser et al., on the other hand, genetically engineered mice to lack the gene for synthesizing 6′-SL in the mammary gland, resulting in milk theoretically devoid of 6′-SL, but the levels in milk were not directly measured [64]. By cross-fostering wildtype (WT) pups to KO dams, the effect of consuming milk with reduced 3′-SL or 6′-SL diets during lactation was investigated in adulthood. The cognitive outcomes of the KO offspring reared in KO or WT dams were also evaluated to differentiate the effects of genetic deficiencies versus HMO reduction in milk [63]. Four groups were compared for behavioral outcomes: CTRL, WT offspring receiving WT milk; MILK, WT offspring receiving SL deficient milk; GENE, KO offspring receiving WT milk; and GENE + MILK, KO offspring receiving SL deficit milk [63]. WT pups that received milk with decreased 3′-SL showed a significant reduction in spatial memory, attention, NOR recognition memory, and altered hippocampal long-term potentiation compared to WT pups that received WT milk. In addition, the KO pups (both receiving WT milk and 3′-SL poor milk) exhibited impairment in spatial memory and general locomotion compared to CTRL mice. These results suggest that 3′-SL exposure in early life has long-term implications for cognitive functions [63]. 

Secondly, in the 6′-SL KO mouse model developed by Hauser et al. [64], the effects of 6′-SL deficiency were observed. After eye-opening, 53 genes involved in the formation and patterning of neuronal circuits were downregulated in WT and KO pups consuming 6′-SL deficient milk. Further, these mice had impaired recognition, sensorimotor gating, and LTP in adulthood, suggesting that the absence of SL during lactation may result in poorer cognitive function throughout life [64]. 

The third gene knock-out study from the same research group engineered double knock-out mice with deletion of genes synthesizing both 3′-SL and 6′-SL to investigate further the effects of reduced SL exposure on cognitive capabilities later in life [68]. As a secondary aim, they evaluated whether exogenous supplementation of 3′-SL and 6′-SL (compared to H_2_O) would counteract the influences caused by the deficiencies in a second experiment. As expected, the concurrent deficiencies of 3′-SL and 6′-SL during lactation resulted in impairments in memory and attention functions, consistent with the previous two studies [63,64]. However, interestingly, in the second experiment, the researchers failed to reproduce the same phenotypic impairments in the WT pups receiving SL-poor milk (MILK group) and H_2_O supplementation, as observed previously in the MILK group in their first experiment. Therefore, they were not able to conclude whether or not exogenous supplementation of MOS to mice that are receiving SL deficit milk would compensate for the observed neurocognitive deficits [68]. The researchers proposed that the failure to replicate these phenotypic impairments in the MILK group may have potentially resulted from the supplementation procedure introducing confounding in behavioral performances, warranting future studies. 

Together, these studies in knock-out mice demonstrate the importance of exposure to milk-borne 3′-SL and 6′-SL during lactation for long-term cognitive and executive functioning in multiple domains, including spatial memory, recognition memory, attention, and synaptic plasticity. 

### 3.4. Fucosyllactose and Cognition

#### 3.4.1. Piglet Models

As noted above, 2′-FL is a predominant HMOS produced by secretor gene-positive mothers. 2′-FL contains fucose, a component of glycoconjugates in the brain [83], supporting a potential role for dietary 2′-FL in cognitive development. Three intervention trials explored the effects of 2′-FL supplementation on cognitive outcomes using piglet models (Table 5).

All three studies used male pigs fed a commercial sow milk replacer as the control diet and the control diet, plus exogenous oligosaccharides for the experimental diets. Sutkus et al. compared the effects of a control diet to 2′-FL- and prebiotic *B. infantis* Bi-26-supplemented diets on the gut-brain axis [67], while Fleming et al. conducted one study that assessed the impact of dietary oligofructose (OF) in combination with 2′-FL on brain development [60], and another study that assessed the impact of HMOS (2′-FL + LNnT), BMOS, and their combination on behavioral outcomes and brain structures [61]. Dietary OF is a non-HMO oligosaccharide of vegetable origin, which has been reported to impact brain-derived neurotrophic factor expression. HMO and non-HMO oligosaccharide benefits for cognitive development were evaluated individually and in combination (OF + 2′-FL). BMOS, on the other hand, are bovine milk-derived oligosaccharides that are less complex in structures but are structurally identical to those found in HMOS, such as 3′-SL and 6′-SL [84]. Supplementation of BMOS to infant formula has been reported as safe and may rescue deficits in weight, height, and head circumference measurements observed in formula-fed infants compared to breastfed infants [84].

Both Sutkus et al. [67] and the two studies by Fleming et al. [60,61] utilized the NOR test to investigate the recognition and working memory of the pigs. These studies found that 2′-FL, OF, and BMOS influenced recognition memory, as shown by increased object visits. However, the type of OS supplementation yielded varying results on recognition memory. Specifically, OF alone increased recognition memory after a 1 h delay, while OF+2′-FL increased recognition memory after a 48 h delay [60]. On the other hand, BMO supplementations (BMOS alone and BMOS+HMO) showed a lesser increase in distance moved per minute during the habituation phase in the NOR task compared to pigs fed no BMOS. HMOS supplementation of 2′-FL and LNnT increased recognition memory after the 1 h delay, but only HMOS in combination with BMOS was associated with increased recognition memory after the 48-h delay. As for the control group, no recognition memory was displayed either after a 1 h or 48 h delay [61]. These results suggest that the combination of HMOS and other oligosaccharide supplementation may result in longer recognition memory retention. 

These studies also explored the effects of MOS supplementation on brain structures, which were investigated using structural MRI. Sutkus et al. [67] observed an increase in relative volume in the pons region of the 2′-FL-supplemented pigs compared to the control. Fleming et al. [60], however, did not find significant alterations in the brain structures, but trending effects (0.05 < *p <* 0.10) on absolute volumes of several brain regions, including olfactory bulbs and thalamus, were found between diets. When looking at HMOS and BMOS and their effects on brain structures, the results suggested that all diet groups with HMOS supplementation displayed larger relative and absolute volumes of cortices and corpus callosum [61].

#### 3.4.2. Rodent Models

Four studies orally administered 2′-FL during lactation to investigate relationships with cognitive development in rodent models. Unlike the studies conducted in piglet models, the studies conducted in rodent models did not perform brain imaging but did conduct behavioral assessments (Table 6). 

Oliveros et al. supplemented 2′-FL to rats during lactation, finding that 2′-FL supplementation resulted in significantly increased performance in NOR, Y maze, and long-term potentiation in their offspring at one year old [53], suggesting the long-lasting effect of 2′-FL exposure in early life, which is consistent with previous supplementation effects of 3′-SL and 6′-SL in rodents. One study tested the effects of combined SL and FL supplementation on the mesolimbic dopamine system in rats. The results showed that the HMOS supplementation exerted sex-dependent effects; 3′-SL + 2′-FL fortification decreased dopamine transporter expression in the ventral tegmental area and increased leptin expression in the nucleus accumbens in females only [62]. This study not only highlighted the impact of HMOS supplementation on the dopamine system but suggested for the first time that variations in response to the HMOS supplementation may be sex-dependent [62]. 

To investigate the beneficial effects of 2′-FL on the gut-brain axis and cognition in obesity, Lee et al. [59] conducted a study on high-fat diet-induced obese mice. They found that 10% 2′-FL supplementation resulted in compositional changes to the gut microbiota and improved gut-brain signaling [59]. Lower 2′-FL doses (1, 2, or 5%) did not significantly affect microbiota composition or attenuate inflammation induced by a high-fat diet, suggesting a dose-dependent effect of these HMOS. 

Another laboratory study investigated the effects of 2′-FL supplementation with both low-fat (control) and high-fat diets on the gut-brain axis [66]. The 10% 2′-FL supplementations reduced intestinal para and transcellular permeability and restored the vagally-mediated gut-brain signaling in mice fed the high-fat diet compared to the low-fat diet. Thus, this study revealed potential new roles for 2′-FL in controlling gut-barrier function and gut-brain signaling during metabolic stress in high-fat-fed mice [66].

### 3.5. Human Studies on HMOS and Cognition

The current literature on the effects of HMOS on cognitive outcomes in human infants is limited compared to data from preclinical models and includes only observational trials. After the selection process, only eight studies involving human subjects were retained in the review (Table 7). The studies applied various cognitive measures and observed mixed associations between HMOS concentrations and cognitive outcomes.

Berger et al. [69] investigated the relationship between 19 HMOS concentrations in human milk and cognitive development in Hispanic infants (*n =* 50) living in Los Angeles, California. This observational study collected human milk samples at 1- and 6-months postpartum and measured cognitive development using the Bayley Scales of Infant Development, third edition (Bayley-III) at 24 months-of-age. The Bayley III was administered by trained personnel to measure the functions of cognitive, language, and motor skills. In the study, only the age-standardized scores for cognitive development were utilized as the dependent variable. The concentration of 2′-FL in milk at one month postpartum was associated with higher infant cognitive development scores at 24 months of age, whereas milk disialyllacto-N-tetraose (DSLNT) concentration at one month was associated with lower cognitive scores at 24 months of age. 2′-FL concentration from milk collected at six months postpartum was no longer related to 24-month cognitive scores. However, several other HMOS concentrations, lactose-N-hexaose (LNH) and fucosyllacto-N-hexaose (FLNH) were related to greater, while LSTb related to lower cognitive development scores at 24 months [69]. This study corroborates findings for the beneficial effects of 2′-FL exposure during lactation for cognitive development in preclinical studies. It implies the potential relevance of other fucosylated and sialylated HMOS for cognitive development. Additionally, associations between HMOS levels of 1- and 6-months were found with child cognitive outcomes at 24 months of age, suggesting sustained effects of early-life HMOS exposure on child development. 

Another study by Jorgensen et al. [72] conducted in Malawian mother-infant pairs (*n =* 659) also collected milk samples at six months postpartum, observing mixed relationships between HMOS structures and later child development, including motor and language skills. These findings were mostly secretor status dependent. For example, in infants born to secretor mothers only, the relative abundances of total fucosylated and total sialylated HMOS were positively associated with infant language ability at 18 months. Infants of secretor mothers with milk samples containing a relative abundance of fucosylated HMOS above the median also showed greater vocabulary at 12 months old than those below the median. On the other hand, positive associations between the relative abundance of sialyllacto-*N*-tetraose b (LSTb) and working memory and executive function were only observed in non-secretors [72]. 

While Berger et al. reported LNH concentrations were positively related to greater cognitive development scores at 24 months [69], Jorgensen et al. instead found a negative association between LNH relative abundance and language at 18 months, but only in infants born by secretor mothers [72]. The lack of consistency in the findings regarding the association between MOS and child development highlights the complexity of the relationship. These inconsistencies may arise from various factors, including maternal secretor status, differences in sample populations, methodologies, outcome measurements, and potential confounding variables. It is important to consider that child development is a multifaceted process influenced by a wide range of genetic, environmental, and nutritional factors, not solely dependent on milk oligosaccharides.

Another observational study of children 2-25 months old (*n =* 99) quantified eight HMOS in milk collected at each study visit [70]. Positive associations between 3′-SL concentrations were observed with receptive and expressive language functions, supporting a higher composite score of early learning criteria, as measured by Mullen Scales of Early Learning. However, the positive association was only observed in mothers with alpha-tetrasaccharide (A-tetra +) in milk, which has been suggested to only be present in mothers with blood type A [70]. This finding is consistent with the study of Malawian children mentioned previously, which found that infants of secretor mothers with higher 6-month total relative abundances of sialylated HMOS had higher language skills at 18 months [72]. Thus, these data indicated the beneficial effects of sialylated HMOS on infant language development. Further, relationships between sialylated HMOS and language may be confounded by maternal genetic background, warranting further investigations. 

Two studies [73,74] measured neurodevelopmental outcomes with the Ages and Stages Questionnaire (ASQ), which measures five domains of development: communication, gross motor skills, fine motor skills, problem-solving, and personal-social skills [85]. Ferreira et al. studied full-term infants and utilized a version of the ASQ tailored for the Brazilian children population [73]. They reported that infants of mothers with lower median HMOS concentrations had a higher risk of various developmental inadequacies at one month of age. For example, a lower 3-FL concentration was associated with the risk of inadequate communication skill development; a lower FLNH concentration was related to inadequate fine motor skills. However, after adjusting for multiple comparisons, only an inverse relationship between LNT concentration and risk for personal-social skill inadequacies remains significant in total samples and in secretor mothers only [73]. The second was an exploratory study in preterm infants that assessed associations between HMOS in milk collected from birth to 7 weeks and infant neurocognitive outcomes measured at two years of age. This is the only study investigating the beneficial effects of HMOS on cognitive development in preterm infants. Aside from receiving their mother’s milk with standardized fortification, the preterm infants were on parental nutrition, and donor milk was used on rare occasions to ensure adequate nutrition. Only Lacto-N-fucopentaose III (LNFP III) concentration and total ASQ scores were positively related in infants of secretor mothers, which was not reported by other studies before [74]. This study extends the relevance of HMOS for cognition and its possible dependence on maternal genetic factors, such as the secretor genes, to preterm infants. 

While there are numerous outcomes of infant cognitive development, assessing executive function in children has become increasingly prevalent [86] due to its critical roles in academic achievement [87], self-regulation and the development of social and cognitive competencies [88]. The core domains of executive functioning include working memory, attention control, cognitive flexibility, and inhibitory control [89]. Several studies in this review explored the relationships between HMOS and executive functioning. Consistent with findings from preclinical studies, a recent study in the Netherlands reported concentrations of 2′-FL and total fucosylated HMOS in the first 12 weeks being related to better parent-reported executive functioning at three years of age in exclusively breastfed infants [76]. This study contributed further evidence of 2′-FL acting as a crucial component for cognitive outcomes. Of note, this study also reported that higher levels of sialylated HMOS were associated with worse executive functioning, but only in partially breastfed infants [76]. To account for formula intake in partially breastfed infants, Willemsen et al. corrected the HMO concentrations by the human milk intake. For example, if the infant received 30% formula, the HMO concentrations will be multiplied by 0.7 [76]. However, among the other studies that included mixed-fed infants [70,73,76], the amount of formula intake was not adjusted in the analysis. Also, two studies [71,72] did not provide information on the mode of feeding. This limitation potentially introduces confounding effects and hinders the accurate interpretation of the observed associations between HMOs and cognitive outcomes.

One study from Spain recruited lactating women with overweight, obesity, or gestational diabetes in pregnancy to investigate potential confounding factors related to maternal health status [71]. Maternal weight status and gestational diabetes did not impact the HMOS levels in milk. However, concentrations of 6′-SL at one month postpartum were positively associated with infant cognitive and motor scale scores at 18 months of age; additionally, 2′-FL was positively correlated with motor scale scores at six months old. Collectively, 6′-SL and 2′-FL levels are linked to better language and motor skills in infants, consistent with findings in preclinical animal models discussed previously [71].

All the human studies discussed above used standardized behavioral assessment questionnaires to evaluate the cognitive development of infants later in life. Although the surveys are standardized, the results were mostly obtained through parent reports, which may not be sensitive enough to detect subtle neurodevelopment variations in infants [74]. However, one study included in the review explored the effects of HMOS exposure on infant brain tissue organization using MRI scanning, and the study was done in the same cohort of infants from Los Angeles [69]. Berger et al. [75] reported that fucosylated and sialylated HMOS were differentially associated with the microstructure of numerous brain tissues [75]. Specifically, the 2′-FL concentration at one month postpartum is associated with greater MD values in the posterior cortical gray matter, posterior white matter, and subcortical gray matter nuclei; 2′-FL exposure was also inversely associated with regional cerebral blood flow (rCBF) and fractional anisotropy (FA) throughout much of the cortical mantle. 3-FL and 3′-SL exposure exhibited differential effects, where 3-FL and 3′-SL concentrations were negatively associated with MD values and positively associated with FA values and rCBF in the white matter throughout the brain [75]. 

## 4. Discussion

The purpose of this narrative review was to summarize current evidence for the impact of MOS consumption in early life on the neurocognitive and brain developmental outcomes in both preclinical models and human subjects. Most studies assessed the effects of milk oligosaccharide supplementation in piglet or rodent models. However, observational analyses conducted in mother-infant pairs assessed associations between human milk HMOS content and cognitive outcomes, providing additional evidence that supports the findings from animal model interventions.

More than 200 individual HMOS structures are reported in human milk [16], but only a few HMOS have been tested in clinical studies. Among the 26 studies in the current review, the preclinical model studies have only investigated the influence of SL or FL supplementation or SL gene knockout on brain and cognitive development. On the other hand, analyses conducted in human subjects focused on the associations between the absolute concentrations or relative abundances of the most abundant HMOS and infant cognition. Beyond SL and FL that were investigated in preclinical models, significant associations have also been observed between various infant learning and memory outcomes and the less abundant HMOS, including LNH [69] and LNFP III [74]. Some HMOS, such as DSLNT and LSTb, was reported to have a negative effect on infant cognitive development [69]. This suggests that individual HMOS with different structures might have differential effects on infant brain development. Given the scarcity of research on less abundant HMOS, further investigation is warranted, especially through well-designed preclinical trials or well-controlled observational studies.

### 4.1. Sialylated MOS and Cognition

Human milk provides infants with about 20% more SA compared to formula [90]. Various studies have shown that the SA component of HMOS is crucial for facilitating infant brain development [77,91]. SA is significantly more abundant in neural cell membranes than other types of membranes, suggesting that SA plays a distinct role in the structure of neural cells [92]. The function of the brain in learning and memory appears to be related to the high recognition abilities of the glycoproteins in the brain [29], and there is evidence indicating that the SA content of brain glycoproteins is involved in memory formation [93,94]. Given that around 70–83% of SA in human milk is bound to HMOS, sialylated HMOS can potentially serve as the source of SA for neurologic development [95]. Animal studies revealed that 3′-SL and 6′-SL supplementation during lactation increased cognitive performance [51,52,54,55,57,58,65], suggesting that sialylated HMOS play an essential role in cognitive development by improving recognition and working memory. 

Piglet studies provide evidence for the provision of SA by sialylated HMOS: formula 3′-SL or 6′-SL supplementation increased bound SA concentration in piglet prefrontal cortex, corpus callosum, and cerebellum [52,54] and upregulated expression of genes related to SA metabolism [57]. 

To investigate the individual effect of SL on brain development, genetically modified rodent models were also developed to generate milk deficient in 3′-SL or 6′-SL or both [63,64,68]. Mouse pups who received SL-deficient milk during lactation demonstrated impairments in spatial and recognition memory, suggesting that early life exposure to SL was critical for optimal memory formation.

Aside from SA within HMO, dietary SA found in other milk components may have a beneficial effect on cognitive development as well. One study utilized three-day-old male piglets to investigate the effect of dietary SA on brain growth, learning, and memory, finding that a protein-bound SA, casein glycomacropeptide, enhanced learning performance and increasing the expression of two genes, *ST8SIA4* and *GNE,* related to learning [96]. Likewise, another study suggested that supplementation of isolated dietary SA led to increased cortical ganglioside SA content in developing rats, suggesting that the beneficial effects of sialic acid on cognitive function may be due to its alteration of brain composition to support early brain development [97]. However, there is a lack of evidence on whether SL promotes its cognitive effects through its SA content or indirectly, such as modulating the gut-brain axis. Interestingly, 6′-SL supplementation resulted in a greater positive effect on enhancing learning than free SA supplementation [55]. The underlying mechanisms driving this differential effect remain to be replicated and fully elucidated.

### 4.2. Fucosylated MOS and Cognition

Fucosylated HMOS, especially 2′-FL, are usually the most abundant HMOS in secretor mothers [98]. Many studies included in this review supported the role of 2′-FL and fucosylated HMOS in learning and memory formation processes [53,59,60,61,62,66,67]. This may be explained by the neuroprotective effects of 2′-FL in animal models [99,100], although the exact mechanism for this effect is unclear. LTP is often considered the cellular analog of learning and memory [101] and is widely used to assess synaptic transmission in neurons [102]. An increase in LTP indicates improved synaptic transmission and suggests that neurons are more capable of adapting to new information and retaining it [103]. While multiple brain regions are involved in learning and memory, the hippocampus is particularly critical for memory formation [104]. Most animal studies included in the current review assessed the effects of milk oligosaccharide supplementation on memory through measurement of hippocampal LTP by implanting stimulating and recording electrodes in the hippocampus [55], consistently demonstrating that 3′-SL and 2′-FL may be involved in the maintenance of LTP for rodents in early life [55,63,64]. Oral administration of L-fucose [105] and 2′-FL significantly improved hippocampal LTP memory skills and impacted synaptic plasticity in rodents [106,107]. However, D-fucose or 3-FL infusion [35] did not generate the same improvements as 2′-FL or L-fucose on hippocampal LTP and other measures of memory performance. This may suggest that the benefits of the fucose moiety versus FL for cognition are structurally dependent.

Nonetheless, a recent mouse study examined the role of 2′-FL and fucose on cognition with stable isotope (^13^C) labeling, finding that benefits from 2′-FL intake were not explained by fucose absorption, as fucose did not cross the blood-brain barrier [40]. Thus, the specific role of 2′-FL on cognition outcomes should be further studied. Also, it is worth noting that the direct link between LTP and cognitive performance is not well-established [108]. Although some studies have shown improved LTP in animals following HMOS supplementation, this association has not yet been confirmed in human subjects.

### 4.3. HMOS and Infant Cognition

The effects of HMOS on brain and cognitive development in human infants are sparsely explored, and the participants of these studies are relatively limited to certain geographic locations. Among the eight human observational studies included in the current review, two found positive associations for 2′-FL concentrations [69,76], while two found positive associations for SL (3′-SL and 6′-SL) with cognitive development measured via infant behavioral assessment questionaries [70,71]. One study reported a unique association between LNFP III concentration and cognitive scores in infants born to secretor mothers [74], while another found negative associations between several less abundant HMO levels (DSLNT and LSTb) and cognitive outcomes [69]. The inconsistency observed in these observational studies may be due to several reasons. First, methods of assessing cognitive development and motor skills varied, with most assessments being parent-reported surveys. The use of questionnaires or surveys to assess cognitive development in infants and children may not provide a complete picture of the phenomenon under investigation. This could be due to the complexity of cognitive development processes that involve a multitude of factors [109], including genetics and environment [110], and cognitive outcomes can be largely influenced by parent-child relationships and the home environment for children before the start of school [111]. Besides, only two studies [72,76] looked at the effect of HMO groups, namely total fucosylated and/or total sialylated HMOS concentrations, on cognitive outcomes. Most studies mainly focused on associations between single HMO concentrations and cognitive measures, leaving a significant knowledge gap in understanding their combined influence or synergistic effects on neurocognitive development. Therefore, bioinformatics tools are needed for HMO diversity and cluster analyses. 

While most human infant studies measured HMO concentrations, Jorgensen et al. [72] reported associations between HMOS and infant cognitive outcomes based on the relative abundances of each HMO structure. Although relative abundance measurements offer insight into the proportions of different HMOS in breast milk, they have inherent limitations that must be acknowledged. One significant concern with relying solely on relative abundance is that it does not provide a direct assessment of the absolute concentration of each HMO. In cases where one specific HMO is relatively high in abundance, it may indicate a corresponding decrease in the relative abundance of other HMOS.

Remarkably, only one [76] study estimated the breastmilk intake by infants, which limits the precision of calculating the absolute amount of HMO intake. This becomes especially pertinent when considering the inclusion of formula feeding in the infants’ diet. For infants who are not exclusively breastfed, the amount of HMO consumed will be even less accurate since both human milk and formula intake are rarely collected. To address this limitation, future research should consider implementing a more precise methodology, such as weighing before and after each breastfeeding session or the use of doubly labeled water to determine milk intake. For combination-fed infants, researchers need to carefully report how they account for formula feeding to ensure the results are accurately interpreted. For example, as mentioned previously, the approach used by Willemsen et al. [76] could be implemented.

### 4.4. Potential Mechanisms of MO Functions in Cognition

Only one of the eight observational studies utilized a brain MRI scanning procedure to investigate the associations between HMOS exposure and infant brain microstructures [75]. Significant relationships between HMO concentrations and brain structures were identified, suggesting a role for HMO in brain maturation processes. For example, 2′-FL concentrations at one month were associated with reduced FA and increased MD in the cortical mantle [75]. A previous study suggested that the decline of FA, coupled with an increase in MD, indicates increased dendritic arborization and synapse formation in the cortical region of the brain [112,113]. Since dendritic arborization and synaptogenesis form the structural basis of learning and memory [75,114,115], maintaining their integrity is important for preventing cognitive dysfunction. Thus, the positive association between 2′-FL and cognitive function may be achieved through enhancing dendritic arborization and synaptic formation. In addition, Berger et al. [75] also reported that 3-FL and 3′-SL concentrations at one month postpartum are associated with increased FA and decreased MD in white matter across most brain areas. Studies suggested that the increased FA in white matter is associated with myelination and axon tract development, which are important for cognitive development [75]. The changes in brain microstructures provide invaluable insight into the potential mechanism of HMO to support the structural maturation of the brain. 

Interestingly, a piglet study in which a combination of 3′-SL and 6′-SL was supplemented to formula at concentrations typically found in mature human milk (61-120 days of lactation) significantly increased corpus callosum white matter MD and AD [54]. However, similar effects were not reported by other animal model studies, and the findings are not consistent with the results from the infant observational study [75]. Since an elevation in MD in the white matter may be related to decreased synaptic density, further investigations are needed [116]. Future studies in humans should continue employing more robust markers of cognitive development, such as brain imaging, neuroelectric measurement, and researcher-administrated cognitive tasks, preferably in a longitudinal setting. 

Another confounding factor in human studies of HMO relationships with cognitive outcomes may stem from other factors that influence cognitive and brain development, including genetics, maternal health conditions, and environmental factors [117]. For example, it has been reported that the association between breastfeeding and infant cognition was modified by maternal genetic variants of the fatty acid desaturase (FADS) gene, which is involved in polyunsaturated fatty acid metabolism [118], where children of mothers with lower FADS1 and higher FADS2 activities showed a significant advantage in cognition at 14 months [118]. Other human studies also demonstrate the interaction effects of FADS2 polymorphism on the breastfeeding IQ relationship [119,120], although the directionality was inconsistent. Among the studies included in the current review, two studies [72,74] reported associations between several HMOS concentrations and cognitive outcome measures only in secretor mothers and their infants. However, the detailed genetic information was not obtained for the mothers. Another study reported differential effects of HMOS on cognitive outcomes by A-tetra blood groups [70]. Thus, maternal genetics could potentially impact the functions of HMOS on infant cognitive development. It would be practical to investigate infant genetics and interactions with HMO exposure in early life. 

Other maternal characteristics, such as pre-pregnancy BMI [121], gestational diabetes [122], as well as mode of delivery [123], have been demonstrated to influence HMOS concentrations in human milk. Thus, it is likely that associations between HMOS and infant cognition can be affected by other maternal factors. Few studies included these factors as covariates in their analyses. However, one study included mothers who developed gestational diabetes and mothers with overweight and obese weight status in the study design [71] but reported no effects of these conditions on the relationships between HMOS and infant cognition when considering pre-pregnancy BMI and diabetic status as covariates [71]. Future clinical trials on HMO and cognition should explore maternal and child genetics, maternal health status, mode of delivery, and other environmental factors as potential confounders. 

Additionally, most of the studies were conducted on full-term animal models or human infants, with the exception of one study of preterm pig models [57] and an exploratory analysis on preterm infants [74]. The results suggested that SAL supplementation to preterm pigs ameliorated deficiencies observed in making correct choices in spatial T-maze navigation, such that they performed similarly to term pigs [57]. Even though the supplementation was bovine milk oligosaccharide-enriched whey with SL, the 3′-SL and 6′-SL content still played an essential role in supporting cognitive functions in preterm pigs. The study on preterm infants did not compare differences between term and preterm infants in their responses to HMOS in human milk. However, they observed greater LNFP III concentrations in human milk related to better ASQ scores in preterm infants, corresponding to five domains (communication, gross motor, fine motor, problem-solving and personal-social skills) [74]. Thus, it is important to further examine this relationship, particularly through controlled clinical trials in preterm infants, as they may be at higher risk for developmental delays and cognitive impairments [124]. Aside from potential cognitive benefits, preterm infants reap numerous benefits from human milk consumption [125], and HMOS are thought to play important roles; infants fed human milk demonstrated a reduced incidence of viral and nosocomial infections compared to formula-fed preterm infants [126]. 

Although the mechanisms by which MOS promote cognitive function are not yet fully understood, several studies suggested that the beneficial effects of MOS occur via modulation of gut microbiota. It has been proposed that differences in microbial colonization patterns and microbiome composition between breastfed and formula-fed infants are largely driven by HMOS [127]. Likewise, it has been reported that bifidobacterial colonization was delayed for infants consuming HM from non-secretor mothers, who lack the enzyme for making 2′-FL compared to secretor mothers [128]. Thus, variations in HMOS composition likely contribute to the differences in *Bifidobacterium* colonization early in life [127], an interaction that may represent a mechanism by which HMOS promote cognitive development. Savignac et al. discovered that supplementation with = *B. longum* 1714 = produced a positive effect on cognition in male mice [129].

Further, in human infants, Carlson et al. conducted a cluster analysis and identified associations between microbiome and cognitive measurements [130]. In addition, upon the investigation of the metabolite fate of ^13^C-labelled 3′-SL (^13^C-3′-SL) and ^13^C-N-acetylneuraminic acid (^13^C-Neu5Ac) in mice, Galuska et al. [131] claimed that ^13^C-Neu5Ac is taken by the gut epithelial cells and not incorporated in the brain. They proposed that gut microbiota is involved in the metabolism of 3′-SL and sialic acid. Together, these findings support the brain–gut–microbiome axis. However, most current studies on the effects of prebiotics on the gut-brain axis are descriptive and limited to investigating indirect influences of prebiotics on brain physiology and behavior. However, there is still a lack of comprehensive understanding of the mechanisms [132]. 

Wang et al. [133] investigated the modulation effect of BMOS and HMOS on the gut microbiota composition in the same pig models from Fleming et al. [61], and they found that BMOS supplementation altered the relative abundance of bacterial taxa in both ascending colon and feces. Specifically, *Bacteroides* abundance was increased by BMOS and BMOS+HMO supplementations, and HMO alone increased the proportion of several taxa, such as *Blautia*. Taken together with the behavioral outcomes, where the pigs with BMOS+HMO supplementation showed long-term recognition memory and increased volumes in the cortices and corpus callosum, suggested that BMOS and HMO could play a role in microbiota composition and cognition. To elucidate the mechanism between gut-brain signaling in the context of HMOS, mediation analyses of the existing data were performed by Fleming et al. [134]. The mediation analysis revealed mediators between gut microbiota, cognitive functions, and brain structures in young pigs, including hippocampal genes related to myelination and neurotransmitters. They pointed out that mediating variables between the gut and brain varied with oligosaccharide intake but in a similar pattern [134]. Therefore, the relationship between HMO consumption, gut microbiome, and cognitive needs additional investigation [134].

Another area for further research is the potential long-term effects of early-life HMOS exposure on cognitive development in children, as long-term benefits of breastfeeding on cognitive development have been demonstrated [135,136,137,138]. Most studies in our review focused on early-life cognitive development, but the potential effects of HMOS may extend beyond infancy and early childhood. In several animal model studies, the beneficial effects of HMOS supplementation are detected through adulthood [53,55,63]. Current data available for cognitive development assessment of human infants ranges from 1 month- to 3 years of age.

In our review, some studies did not account for multiple comparisons [69,71], raising concerns about the risk of Type 1 errors [42]. With numerous HMOS tested and multiple cognitive measures explored, the probability of obtaining false-positive results increases. To address this issue, researchers should apply appropriate statistical corrections to ensure the reliability of findings. Transparent reporting and replication of significant results across independent studies will strengthen the validity of associations between HMOS and cognitive outcomes.

While the collective results from preclinical models are promising, another significant concern for animal studies is the risk of publication bias, where the studies with positive results are more likely to be published [139]. This bias can lead to an overrepresentation of positive outcomes in the literature, potentially skewing the overall perception of the effects of HMOS on neurodevelopment. Thus, clinical studies on mother-infant pairs are needed in combination to fully understand the role of HMOS in cognitive development in humans. Current evidence suggests the benefits of several individual HMOS components and total sialylated or fucosylated HMOS for improving cognitive development and brain maturation. Routine supplementation of HMO in the formula may benefit infants that cannot be breastfed [140], although more controlled clinical trials are necessary before this can be widely applied. 

## 5. Conclusions

The results from this review demonstrate a consistent link between early life HMOS consumption and cognitive developmental outcomes, including motor skills, language development, working and reference memory, and IQ. Although most of the relationships were correlations and non-causal, 2′-FL, 3-FL, 3′-SL, and 6′-SL were consistently shown to provide a supportive role in brain and cognitive outcomes. These results underscore the potential importance of these specific HMOS in promoting optimal cognitive development in early life. This review highlights the need for clinical studies investigating the mechanism by which MOS promote learning and memory formation in infants. For example, longitudinal studies with neuroimaging components may be informative in shedding light on the neural basis of HMO-related effects on cognitive development.

## Figures and Tables

**Table 3:** Sialyllactose and cognitive outcomes in preterm and term pigs.

Gestation	Diet	Analyses	Outcome	Ref.
Preterm piglets; 90% gestation (day 106) & Term piglets; C-section & Term piglets; Vaginally delivered	PRE-CON: Raw BM + 6g/L lactose PRE-SAL: Raw BM + 8.5 g/L SAL-10 (380 mg SL/L) TERM-CON: Raw BM + 6 g/L lactose TERM-NAT: Naturally BF	Motor acquisition Home cage activity Open filed assessment Spatial T-maze Right cerebral hemisphere MRI Hippocampal gangliosides SA quantification qPCR hippocampal gene expression In vitro computer-assisted fluorescence microscopy with H_2_O_2_ challenge for neuronal survival analysis	PRE-SAL group had a higher (*p <* 0.05) percentage of pigs reaching the learning criteria of T-maze compared to PRE-CON pigs. PRE-CON group spent a longer time (*p <* 0.01) on decision-making compared to TERM-CON pigs. Hippocampal SA did not differ (*p* > 0.05) between PRE-CON and PRE-SAL pigs. Hippocampal genes associated with myelination (*p <* 0.05) and SA metabolism (*p <* 0.05) were upregulated in PRE-SAL groups compared to PRE-CON pigs. In vitro SAL and lactose treatment reduced cell death and promoted neuronal survival compared to control neurons (*p <* 0.05).	[57]
Term pigs; PND2	CON: formula for nutritional needs of growing pigs SAL: CON + SAL-10 (380 mg SL/L)	NOR for object recognition memory Accelerometers for home-cage activity analysis	No difference in recognition memory was found between SAL and CON (all *p* ≥ 0.11). No difference in diurnal activity between SAL and CON (all *p* ≥ 0.56)	[56]
Term pigs; PND2	CON: formula for nutritional needs of growing pigs LOW: CON + bovine-derived modified why enriched with SL(SAL-10) (130 mg SL/L) MOD: CON + SAL-10 (380 mg SL/L) HIGH: CON + SAL-10 (760 mg SL/L)	Right hemisphere SA quantification (hippocampus, cerebellum, and prefrontal cortex) Whole brain structural MRI Whole brain MRS for brain metabolite quantification DTI for white matter maturation and axonal tract integrity Fractional anisotropy (FA) Axial diffusivity (AD) Mean diffusivity (MD) Radial diffusivity (RD)	No differences in total and free SA between treatments (*p* > 0.05). Bound SA in the prefrontal cortex was higher in CON and HIGH groups compared to LOW and MOD groups (*p* = 0.05). Free SA-to-bound SA in the hippocampus was higher in the MOD and LOW groups compared to the CON and HIGH groups (*p* = 0.04). Corpus callosum MD (*p <* 0.01) and AD (*p <* 0.01) were higher in the MOD group compared to other groups.	[54]
Term pigs; PND3	CON: SMR SL: CON + 3′-SL (7.6 g/kg) and 6′-SL (1.9 g/kg) SL/SLN: CON + 3′-SL (7.04 g/kg), 6′-SL (1.74 g/kg), and 6′-sialyllactosamine (0.72 g/kg)	3T MRS for brain neurotransmitter and metabolites concentrations (Whole brain, cerebrum, and cerebellum)	SL/SLN increased (*p <* 0.05) absolute and relative amounts of myo-inositol (mlns) and glutamate + glutamine (Glx) Positive correlations between 3′-SL and SLN intake and brain metabolite Glu (*p* = 0.017), mlns (*p* = 0.013) and Glx (*p* = 0.032) levels in SL and SL/SLN groups	[58]
Term Göttingen minipigs; 2 weeks old	CON: Milk replacer with no additional oligosaccharides FN: CON + 4 g/L mixture of fucosylated (2′-FL+di-FL) and neutral (LNT + LNnT) oligosaccharides SL: CON + 0.68 g/L sialylated (3′-SL + 6′-SL) oligosaccharides FN + SL: CON+ 4 g/L FN + SL After weaning: high-energy, pelleted, obesogenic diet for all groups	Behavioral procedure tasks Spatial hole board Open field Novel object exposure Runway Single-feed test for appetite measurement Home pen behavior observation	SL group improved reference memory (*p* ≤ 0.010) between 16 to 29 weeks compared to CON. FN, SL, and FN+SL had longer trial durations (*p <* 0.05) compared to CON, between 16 to 29 weeks and 39–45 weeks. FN group spent the most time displaying ingestive behaviors between 0 to 11 weeks (*p <* 0.05)	[65]
Term pigs; PND1	CON: formula adjusted for nutrient requirements of neonatal pigs CON + 2 g 3′-SL/L CON + 4 g 3′-SL/L CON + 2 g 6′-SL/L CON + 4 g 6′-SL/L CON + 2 g PDX/L + 2 g GOS/L	Left hemisphere SA analysis (cerebral cortex, cerebellum, corpus callosum, and hippocampus) Microbiota quantification	2 g 3′-SL and 6′-SL/L increased (*p* ≤ 0.05) total SA concentration in the corpus callosum by 15% compared to CON 4 g 3′-SL increased (*p* ≤ 0.05) total and ganglioside SA concentration in the cerebellum by 10% compared to CON Quadratic effect of dose for 3′-SL and 6′-SL in total and ganglioside-bound SA (*p* ≤ 0.05) Significant differences between proximal (*p* = 0.001) and distal colon (*p* = 0.009) microbiota of piglets fed the 4 g 6′-SL/L and CON Significant differences between proximal (*p* = 0.006) and distal colon (*p* = 0.032) microbiota of piglets fed the 2 g PDX/L + 2 g GOS/L and CON	[52]

Abbreviations: BM, bovine milk; BMO, bovine milk oligosaccharide; BF, breastfeeding; DTI, diffusion tensor imaging; FN, fucosylated and neutral oligosaccharides; GOS, galactooligosaccharides; MRI, magnetic resonance imaging; MRS, magnetic resonance spectroscopy; PDX, polydextrose; SL, sialyllatose; SA, sialic acid; SMR, sow milk replacer.

**Table 4:** Sialyllactose and cognitive outcomes in rodent models.

Gestation	Diet	Analysis	Outcome	Ref.
Mice; PND 0	Genotyping WT: wild type KO: knock-out for the gene synthesizing 3′-SL Diets CTRL: WT offspring with 3′-SL in milk MILK: WT offspring with reduced 3′-SL in milk GENE: KO offspring with 3′-SL in milk GENE + MILK: KO offspring with reduced 3′-SL in milk	NOR T-maze Spontaneous Alternation Test Elevated 0-maze for anxiety-relate behavior. Pre-pulse inhibition Barnes maze Attentional set-shifting task Sucrose preference General locomotion Glucose tolerance test Electrophysiology Experiments	MILK (*p* = 0.025), GENE (*p <* 0.05), and GENE+MILK (*p <* 0.05) mice had decreased spatial memory compared to CTRL mice MILK mice had reduced (*p <* 0.05) attention and higher response to glucose injection compared to other groups MILK mice had reduced (*p* = 0.03) number of spontaneous alternations compared to CTRL MILK mice had reduced (*p <* 0.05) recognition memory compared to CTRL and GENE + MILK LTP was lower in GENE + MILK (*p <* 0.05) and CTRL (*p <* 0.05) mice compared to GENE LTP was lower (*p <* 0.05) in GENE + MILK mice compared to MILK	[63]
Mice; 6–8 weeks old	CON: AIN-93G semi-purified laboratory mouse diet 6′-SL: CON + 5% 6′-SL 3′-SL: CON + 5% 3′-SL	Social disruption stressor (SDR)-induced anxiety-like behavior assessment. Open field and Light/Dark preference test ELISA for serum corticosterone Microbiota sequencing Brain cell proliferation and immature neuronal assessment	CON mice had higher (*p <* 0.05) microbiota Shannon Diversity Index compared to 6′-SL mice 6′-SL and 3′-SL mice had altered microbial beta-diversity compared to CON mice (*p <* 0.01) 6′-SL and 3′-SL mice resulted in significant taxonomic shifts at phylum and genus levels (*p <* 0.05) SDR changed microbial beta-diversity in CON mice (*p <* 0.05) SDR did not induce shifts in microbial beta-diversity in 6′-SL (*p* = 0.138) and 3′-SL (*p* = 0.077) mice 6′-SL and 3′-SL mice spend less time in dark zone compared to CON mice under SDR (*p <* 0.05) 6′-SL mice significantly decreased proliferation within the hippocampus compared to CON regardless of SDR (all *p <* 0.05) 6′-SL and 3′-SL mice rescued reduction of immature neurons induced by SDR compared to CON (all *p <* 0.05)	[51]
Mice; PND 0	Genotyping WT: wild type KO: knock-out for the gene synthesizing 6′-SL Diets CTRL: WT offspring with 6′-SL in milk MILK: WT offspring without 6′-SL in milk GENE: KO offspring with 6′-SL in milk GENE + MILK: KO offspring without 6′-SL in milk	Maternal behavior assessment Fox scale for Neurodevelopmental milestones NOR T-maze test PPI Barnes maze General locomotion Attentional set-shifting task for executive functions Electrophysiology experiments for LTP assessment Gene expression analyses Metabolomics Microbiota analyses Brain Neu5Ac quantification	MILK and GENE + MILK mice increased (*p* = 0.015) general locomotion in adulthood Only CTRL mice showed a preference for novel objects in the NOR test. MILK mice had impaired spatial memory retention compared to CTRL mice (*p* = 0.02) MILK mice required more trials for the CD phase compared to CTRL (*p* = 0.0218) and GENE (*p* = 0.0041) GENE + MILK mice required more trials for the CD phase compared to GENE (*p* = 0.023) MILK mice required more trials for the IDS phase compared to CTRL (*p* = 0.003) and GENE (*p* = 0.017) MILK mice required more trials for the EDS phase compared to CTRL (*p* = 0.008), GENE (*p* = 0.0005), and GENE+MILK (*p* = 0.006). CTRL and GENE mice showed PPI, but MILK and GENE + MILK mice failed to exhibit PPI MILK mice had increased LTP compared to CTRL mice (*p* = 0.04) At eye-opening, MILK mice had a downregulation of 53 genes involved in neuronal circuits formation and patterning compared to CTRL	[64]
Rats; PND 3	CON: BF + water Neu5AC: BF + free Neu5AC to mimic natural Sia level in rat milk 6′-SL: BF + free 6′-SL to mimic natural Sia level in rat milk	HPLC for SA content determination Western blotting In vivo LTP measurement Classical behavioral tests NOR Y maze with blocked arm IntelliCage^®^ Protocol for spontaneous and learning behavior	6′-SL rats showed more PSA-NCAM in the frontal cortex at weaning compared to Neu5AC (*p =* 0.012) and CON (*p =* 0.042) 6′-SL rats had improved LTP in male rats at one year compared to CTRL (*p* ≤ 0.05) 6′-SL (*p =* 0.0352) and Neu5Ac (*p =* 0.0304) rats displayed longer exploration time in NOR at one year Higher percentage of 6′-SL (*p <* 0.0001) and Neu5AC (*p =* 0.0004) rats chose novel arm in the Y maze compared to CON A higher percentage of 6′-SL (*p =* 0.0279) rats chose novel arm in the Y maze compared to Neu5AC rats Higher percentage of 6′-SL (*p =* 0.0483) and Neu5AC (*p =* 0.0237) rats had better performance in IntelliCage^®^ test	[55]
Mice; PND 0	Genotyping WT: wild type dKO: double-knock-out for milk reduced in 3′-SL and without 6′-SL Diets—Experiment 1 CTRL: WT offspring with WT BF MILK: WT offspring with dKO BF GENE: dKO offspring with WT BF GENE + MILK: KO offspring with dKO BF Diets—Experiment 2 CTRL-H2O: WT offspring with WT BF+water MILK-H2O: WT offspring with dKO BF + water CTRL-SL: WT offspring with WT BF + 3′SL–6′SL solution MILK-SL: WT offspring with dKO BF + 3′SL–6′SL solution	NOR T-maze Bernes maze PPI Attentional set-shifting task Glucose tolerance test	Experiment 1: MILK and GENE + MILK groups showed reduced spontaneous alternation in T-maze compared to CTRL and GENE milk (*p =* 0.005) All groups except for the MILK group showed significant preference (*p =* 0.015) All groups showed reduced long-term spatial memory in the Bernes maze compared to CTRL (*p =* 0.01) CTRL and GENE groups showed intact PPI, while MILK and GENE + MILK groups failed to MILK subjects need more trials in the SD and CD phases of the attentional set-shifting task compared to CTRL (*p <* 0.05) Experiment 2: No differences were observed for all behavioral tests between treatment groups.	[68]

Abbreviations: WT, wildtype; KO, knockout; dKO, double-knockout; CON, control; PND, postnatal day; SDR, social disruption stressor; PPI, Prepulse inhibition; ELISA, Interleukin-6 Enzyme-Linked Immunosorbant Assay; SL, sialyllatose; SA, sialic acid; SMR, sow milk replacer; CD, compound discrimination; CDR, compound discrimination reversal; IDS, intra-dimensional shift; EDS, extra-dimensional shift; BF, breastfeeding; SD, simple discrimination; CD, compound discrimination.

**Table 5:** Fucosylated milk oligosaccharides and cognitive outcomes in piglets.

Gestation	Diet	Test	Outcome	Ref.
Term pigs; PND 2	CON: SMR supplemented with 0.532% lactose FL: CON + 0.532% 2′-FL BI: CON + 10^9^ CFU/pig/d Bi-26 FLBI: FL + BI	NOR Structural MRI DTI	FL resulted in a larger (*p =* 0.046) relative volume in the pons region *B. infantis* Bi-26 resulted in smaller (*p <* 0.05) absolute volume in the corpus callosum, left and right internal capsules, left and right putamen-globus pallidus, left caudate, left cortex, lateral ventricles, and medulla; and smaller (*p <* 0.03) relative volume of the left and right putamen-globus pallidus. No effects of FL and Bi-26 on novel recognition memory FL group had a greater (*p <* 0.05) number of familiar object visits in NOR than in CON. BI and FLBI groups spent less (*p =* 0.002) time investigating familiar object	[67]
Term pigs; PND 2	CON: SMR OF: Control + 5 g/L Oligofructose (OF) + 0 g/L 2′-FL OF + 2′-FL: Control + 5 g/L OF + 1 g/L 2′-FL	NOR Structural MRI DTI MRS Hippocampal gene expression	CON failed to show recognition memory after a 1 or 48 h delay. OF showed recognition memory only after 1 h delay (*p <* 0.001) OF + 2′-FL showed recognition memory only after 48 h delay (*p =* 0.001) OF showed higher (*p =* 0.022) novel object visit frequency than CON after 1 h delay OF + 2′-FL had increased (*p =* 0.038) sample object exploration time through trial compared to CON OF and OF + 2′-FL had increased (*p =* 0.019) olfactory bulbs relative volume compared to CON OF showed lower hippocampal mRNA expression of DRD3, GABBR1, HDAC5/8, NCAM1, and CHRM2 (all *p <* 0.045) compared to CON OF + 2′-FL had higher hippocampal mRNA expression of DRD3, GABBR1, HDAC5, and NCAM1 (all *p <* 0.045) compared to CON	[60]
Term pigs, PND 2	CON: SMR BMOS: CON + 12.4 g/L BMOS HMO: CON + 1.0 g/L of 2′-FL + 0.5 g/L of LNnT BMOS + HMO: CON + 12.4 g/L of BMOS + 1.0 g/L of 2′-FL + 0.5 g/L of LNnT	NOR Structural MRI DTI MRS Hippocampal gene expression	HMO showed recognition memory after a 1-h delay (*p =* 0.038) BMOS + HMO showed recognition memory after 48-h delay (*p =* 0.045) CON and BMOS + HMO showed similar absolute and relative volumes of caudate, lateral ventricles and pons as HMO and BMOS HMO and BMOS+HMO had larger relative cortices and corpus callosum compared to BMOS (all *p <* 0.05) HMO and BMOS downregulated many genes in the hippocampus, whereas BMOS + HMO upregulated many of the same genes	[61]

Abbreviations: CON, control; SMR, sow milk replacer; NOR, novel object recognition; MRI, magnetic resonance imaging; DTI, diffusion tensor imaging; MRS, magnetic resonance spectroscopy; PDX, polydextrose; SL, sialyllatose; SA, sialic acid; SMR, sow milk replacer; DRD3, dopamine receptor D3; GABBR1, GABA type B receptor subunit 1; HDAC5/8, histone deacetylases 5 and 8; NCAM1, neural cell adhesion molecule 1; CHRM2, cholinergic receptor muscarinic 2.

**Table 6:** Fucosylated milk oligosaccharides and cognitive outcomes in rodents.

Gestation	Diet	Analysis	Outcome	Ref.
Rats; PND 3	CON: BF + 1 g/kg body weight of water 2′-FL group: BF + 1 g/kg body weight of 2′-FL per day	In vivo LTP at six weeks in the hippocampus for Sprague-Dawley rats In vivo LTP at 1 year for Lister Hooded rats NOR Y maze MWM	2′-FL evoked larger LTP compared to CON (*p <* 0.05) at six weeks and one year 2′-FL spent longer time exploring the novel object than the familiar object (*p =* 0.03) 2′-FL spent longer time exploring objects compared to CON at 1 year (*p =* 0.0475) 2′-FL showed lower latency to the novel arm in the Y maze compared to CON at one year (*p =* 0.0331) 2′-FL had a higher percentage of rats that visited the novel arm as the first choice in the Y maze compared to CON at one year (*p =* 0.0138)	[53]
Rats; PND21	CON: AIN-93G nutritionally complete diet 3′-SL: Control + 0.625% wt/wt 3′-SL 2′-FL: Control + 0.625% wt/wt 2′-FL 3′-SL+2′-FL: Control + 0.625% wt/wt 3′-SL + 0.625% wt/wt 2′-FL	RNA extraction and cDNA qPCR Protein extraction and quantification for NAc and VAT tissue Western blot procedure	3′-SL + 2′-FL females had decreased VAT DAT expression (*p =* 0.032) compared to CON females 3′-SL + 2′-FL females had increased NAc leptin expression (*p <* 0.05) compared to CON females Male CON rats had lower DAT expression (*p =* 0.039) had higher GhrelinR (*p <* 0.05) and leptin (*p <* 0.05) expression than females Male rats had lower NAc leptin expression (*p =* 0.047) than females	[62]
Mice; 6-week-old	LF: 10% kcal as fat research diet HF: 45% kcal as fat research diet HF 1% 2′-FL: HF + 98.4% purity 2′-FL 1% (*w/v*) HF 2% 2′-FL: HF + 98.4% purity 2′-FL 2% (*w/v*) HF 5% 2′-FL: HF + 98.4% purity 2′-FL 5% (*w/v*) HF 10% 2′-FL: HF + 98.4% purity 2′-FL 10% (*w/v*)	CCK sensitivity assessment OGTT RNA extraction and quantitative RT-PCR Immunofluorescence Histology Hepatic lipid accumulation assessment Blood analysis for LPS-bind protein Microbiota DNA sequencing Metabolomic analysis	10% 2′-FL results in less weight gain (*p <* 0.001) with the HF diet compared to the HF 10% 2′-FL decreased food intake (*p <* 0.05) compared to HF 10% 2′-FL suppressed (*p <* 0.01) the increase in fat mass resulting from HF 10% 2′-FL restored the CCK-induced inhibition of food intake for HF mice (*p <* 0.05) 10% 2′-FL resulted in compositional changes in the microbiota (*p <* 0.05) and metabolites (*p <* 0.05) compared to LF and HF mice 10% 2′-FL attenuates the HF-induced inflammation (*p <* 0.05) compared to HF 10% 2′-FL decreased (*p <* 0.05) the upregulation of PPARγ gene expression induced by HF	[59]
Mice; 6-week-old	LF/CON: 10% kcal as fat diet HF/CON: 45% kcal as fat diet LF/2′-FL: LF/CON + 10% 2′-FL (*w/w*) HF/2′-FL: HF/CON + 10% 2′-FL (*w/w*)	Y-maze Open field test for general locomotion NOR OGTT Barrier function assessment RNA extraction and quantitative RT-PCR Immunohistochemistry Histology Hepatic lipid accumulation Microbiota DNA sequencing 16S metagenomic analysis Metabolomic analysis	HF/2′-FL decreased energy intake (*p <* 0.05 at weeks 4–8) and fat mass (*p <* 0.05 at weeks 2,4,6,8) compared to HF HF/2′-FL decreased (*p =* 0.001) the upregulation of PPARγ gene expression induced by HF HF/2′-FL downregulated (*p <* 0.001) the SREBP-1c gene expression compared to LF/2′-FL 2′-FL decreased the size of adipocytes in visceral adipose tissues compared to control for both LF (*p <* 0.01) and HF (*p <* 0.05) group 2′-FL decreased intestinal transcellular permeability (*p <* 0.01) in HF group 2′-FL decreased intestinal para- (*p <* 0.05) and transcellular (*p <* 0.05) permeability in the LF group 2′-FL increased IL-22 gene expression that regulates epithelial homeostasis in the ileum for both LF (*p <* 0.05) and HF (*p <* 0.05) group HF/2′-FL restored vagally-mediated gut-brain signaling integrity compared to the HF group (*p <* 0.05) No detectable effects were found on any cognitive outcome. 2′-FL had different gut microbiota beta diversity compared to LF and HF groups (both *p =* 0.001) 2′-FL significantly shifted microbiota composition and cecal metabolites	[66]

Abbreviations: NOR; Novel Object Recognition; MWM, Morris Water Maze; DA, dopamine; DAT, dopamine transporter; RNA, messenger RNA; NAc, nucleus accumbens; TH, tyrosine hydroxylase; VTA, ventral tegmental area; OGTT, oral glucose tolerate test; PPARγ, peroxisome proliferator-activated receptor gamma.

**Table 7:** HMOS and cognitive outcomes in human observational studies.

Maternal Condition	HMO Assessment	Covariates Adjusted	Tests	Outcome	Ref.
At least partially BF at the study visit	Complete expression of HM collected by pump from the right breast. HMOS quantified with LC with fluorescence detection	Infant age at milk collection Milk collection site and batch Multiple comparisons corrected with Holm-Bonferroni method	S (MSEL) Early learning composite (ELC) score	Positive association (*p =* 0.002) between 3′-SL level and ELC scores of early learning in A-tetra positive group Positive association between 3′-SL level and receptive (*p =* 0.015) and expressive (*p =* 0.048) language scores in A-tetra positive group Interaction effect (*p =* 0.03) between 3′-SL level and age for receptive language scores A-tetra positive group	[70]
Hispanic mothers with pre-pregnancy normal or overweight Exclusively BF for six months	Complete expression of HM collected after 1.5 h fasting by pump from one breast. HMOS quantified with HPLC-MS and internal standards	Maternal secretor status Maternal age at delivery Education level Infant sex Infant age Infant birth weight Not corrected for multiple comparisons	Bayley Scales of Infant Development (Bayley III)	Maternal pre-pregnancy BMI negative predicted (*p =* 0.03) infant cognitive development score 2′-FL at one month (*p ≤* 0.01), LNH (*p ≤* 0.02) and FLNH (*p ≤* 0.02) at six months were associated with higher infant cognitive development score DSLNT at one month (*p =* 0.02) and LSTb at six months (*p <* 0.01) were negatively associated with infant cognitive development scores	[69]
Study groups: Healthy normal weight Overweight Obese GDM No information is available on BF	HM collected before and after each feed throughout one day HMOS quantified with UHPLC-MS/MS and 2′-FL/6′-SL external standards	GWG Maternal IQ Maternal education Study groups Prepregnancy BMI Not corrected for multiple comparisons	Bayley III	Positive association (*p =* 0.041) between 6′-SL and composite cognitive scores in infants at 18 months when adjusted for GWG, maternal IQ and education, and study groups Positive association between 6′-SL and composite cognitive scores (*p =* 0.019) and motor scores (*p =* 0.043) in infants at 18 months when adjusted for prepregnancy BMI and study groups Positive association between 2′-FL and motor scores (*p =* 0.041) in infants at six months when adjusted for prepregnancy BMI and study groups	[71]
Exclusively BF for seven weeks	Complete expression of HM by manual breast pump from one breast HMOS and SA quantified with LC and fluorescence detection	Maternal age Pregravid BMI Self-perceived income Educational level Tobacco use Number of previously breastfed children Course of pregnancy and delivery process Multiple comparisons corrected with Benjamini-Hochberg procedure	Ages and Stages Questionnaire (ASQ)	Positive association (*p =* 0.009) between LNFP-III content and total ASQ scores at two years old for infants born to secretor mothers	[74]
Healthy women 67.0% Exclusively BF at one month	HM collected manually by hand HMOS quantified by HPLC with fluorescence detection and internal standards	Gestational age at birth GWG Prepregnancy BMI Maternal age Parity Mode of BF at one month Multiple comparisons corrected with Benjamini-Hochberg procedure	Brazilian Ages and Stages Questionnaire (ASQ-BR)	Negative associations between: LNT and risk of inadequate development for personal-social skills (HR = 0.06) and ≥2 developmental domains (HR = 0.06) LNT and risk of inadequate development for personal-social skills (HR = 0.09) and ≥2 developmental domains (HR = 0.05) in secretor mothers only	[73]
Women from the iLiNS project No information is available on BF	HM was collected manually for a single full breast. HMOS absolute abundance by nano-LC-chip/time-of-flight MS with standards or relative abundance where standards are not available.	Maternal age Maternal height Maternal BMI Parity Education Food security HIV status Hemoglobin Household assets Residential location Infant sex Season of milk sample collection Family Care Indicator Score (only for developmental outcomes at 18 mos) Child’s mood, activity level, and willingness to interact with the tester (only for motor development/executive function and working memory model) Multiple comparisons corrected with Benjamini–Hochberg method	Motor development (Kilifi Development Inventory Language development (MacArthur–Bates Communicative Development Inventory) Socioemotional development (Profile of Social and Emotional Development) Working memory and executive function (A-not-B task)	Positive association between HMO 5311a and motor skills (*p =* 0.003) Negative association between HMO 5130a and language at 18 months (*p =* 0.002) Positive association between total fucosylated (*p =* 0.007) and total sialyated (*p =* 0.033) HMOS relative abundances and language at 18 months in infants of secretor mothers Negative association (*p =* 0.003) between 6′-SL and walking at 12 months in infants of secretor mothers Positive association (*p =* 0.049) between LNnT and walking at 12 months in infants of secretor mothers Positive associations between F-LSTc (*p =* 0.004) and DFLNnO II (*p =* 0.044) and motor skills at 18 months in infants of secretor mothers Positive associations between DFLNHa (*p =* 0.02) working memory and executive function at 18 months in infants of secretor mothers Positive association (*p =* 0.007) between LSTb relative abundances and working memory and executive function at 18 months in infants of nonsecretor mothers	[72]
Healthy mothers in the Netherlands 71.4% Exclusively BF for 12 weeks	HM collected manually or with a breast pump HMOS quantified by UPLC-MS and HPAEC-PAD	Gestational age at birth Maternal education level Parent(s) executive functioning Sample-to-sample variations Estimated daily milk intake Proportion of human milk feeding Multiple comparisons corrected with Bonferroni adjustments	The Behavior Rating Inventory of Executive Function-Preschool Version (BRIEF-P) Ratings of Everyday Executive Functioning (REEF)	Analyses with exclusively breastfed infants: Higher 2′-FL (*p =* 0.02) and grouped fucosylated HMOS (*p =* 0.03) were associated with higher executive functioning at three years old (REEF) Analyses including partially breastfed infants: Higher levels of grouped sialylated HMOS (*p =* 0.05) were associated with worse executive functioning (BRIEF-P)	[76]
Healthy mothers with full-term singleton birth Exclusively BF at one month	HM collected with a breast pump for a single full breast HMOS isolated with high-throughput SPE and quantified with MS	Prepreganancy BMI Postmenstrual age at the time of MRI scan Infant birthweight Infant sex Multiple comparisons corrected with Benjamini–Yekutieli procedure	MRI DTI ASL	At one month postpartum: Negative associations between: 2′-FL and FA values in the cortex (*p =* 0.001) 2′-FL and rCBF in the cortical gray matter of the frontal, temporal, parietal, and occipital lobes (all *p <* 0.01) 3-FL and MD values in left IC (*p =* 0.007) and posterior white matter (*p <* 0.001) 3′-SL and MD values in the posterior white matter (*p =* 0.007) Positive associations between: 2′-FL and MD values in the posterior cortical gray matter, posterior white matter, and subcortical gray matter nuclei (all *p <* 0.01) 3-FL and FA values in the white matter throughout the frontal, temporal, parietal, and occipital lobes, and left IC and right aCR (all *p <* 0.05) 3-FL and rCBF in the most part of the cortex, the white matter of the frontal, temporal, parietal, and occipital lobes, and subcortical gray matter nuclei (all *p <* 0.05) 3′-SL and FA values in the white matter throughout the brain (*p <* 0.05) 3′-SL and rCBF in the white matter bilaterally (all *p <* 0.01) and in the cortical gray matter of the frontal lobe (*p <* 0.001) 6′SL was not significantly associated with any MRI measures	[75]

Abbreviations: BF, breastfeeding; GDM, gestational diabetes mellitus; GWG, Gestational weight gain; SA, sialic acid; HR, hazard ratio; iLiNS, International Lipid-Based Nutrient Supplements; PGC-UPLC-MS, porous graphitized carbon-ultra high-performance liquid chromatography–mass spectrometry; HPAEC-PAD, high-performance anion exchange chromatography with pulsed amperometric detection; SPE, solid phase extraction; MS, mass-spectrometry; MRI, Magnetic Resonance Imaging; DTI, diffusion tensor imaging; ASL, arterial spin labeling; FA, Fractional anisotropy; MD, mean diffusivity; AD, axial diffusivity; RD, radial diffusivity; rCBF, Regional cerebral blood flow; IC, internal capsule; aCR, anterior corona radiata.

## Data Availability

Not applicable.
